# Editorial: New Advanced Wireless Technologies for Objective Monitoring of Motor Symptoms in Parkinson’s Disease

**DOI:** 10.3389/fneur.2018.00216

**Published:** 2018-04-04

**Authors:** Fernanda Irrera, Joan Cabestany, Antonio Suppa

**Affiliations:** ^1^Department of Information Engineering, Electronics and Telecommunication, Sapienza University of Rome, Rome, Italy; ^2^Technical Research Centre for Dependency Care and Autonomous Living (CETpD), Universitat Politècnica de Catalunya, Vilanova i la Geltrú, Spain; ^3^Sense4Care, Cornellà de Llobregat, Spain; ^4^Department of Human Neuroscience, Sapienza University of Rome, Rome, Italy; ^5^Istituto Neurologico Mediterraneo (IRCCS), Pozzilli, Italy

**Keywords:** inertial measurement unit, wearable sensors, wireless technology, Parkinson’s disease, freezing of gait

**Editorial on the Research Topic**

**New Advanced Wireless Technologies for Objective Monitoring of Motor Symptoms in Parkinson’s Disease**

Nowadays, a growing number of researchers are using advanced wearable technologies with inertial measurement units (IMUs) to improve the evaluation of motor symptoms in patients with Parkinson’s Disease (PD). In this context, wearable sensors are promising technologies possibly helpful for the overall clinical management of PD. The present Research Topic entitled “New Advanced Wireless Technologies for Objective Monitoring of Motor Symptoms in Parkinson’s Disease” explores advances and perspectives of new wearable devices applied to patients with PD to support clinical assessment with objective methods. The 11 manuscripts included in this research topic deal with the evaluation of a wide range of motor symptoms in patients with PD, including the classical cardinal signs, such as bradykinesia, rigidity, tremor, and postural instability, and disabling gait disorders like freezing of gait (FOG) that significantly increase the risk of falls in patients with PD, resulting in a negative impact on quality of life. Accordingly, di Biase et al. examined the best sensor location on patients’ bodies to quantify bradykinesia and rigidity, discriminate patients with PD and healthy subjects and finally distinguish specific motor state (On and Off state of therapy) in PD. Similarly, Spasojević et al. proposed a wireless armband device acquiring electromyography (EMG) and IMU data for quantitative assessment of the arm/hand movements and bradykinesia in PD. The differential diagnosis among various parkinsonian syndromes would also benefit from the objective analysis of motor symptoms through wearable technologies as demonstrated by Bonora et al. These authors used wearable IMU to assess balance in patients with PD and other types of parkinsonian syndromes, as well as in healthy subjects, discriminating specific abnormalities of anticipatory postural adjustments among the three groups. The use of wireless wearable devices also enables long-term monitoring of patients with PD even in a domestic environment, thus overcoming the well-known clinical difficulty of studying motor fluctuations in PD. In this regard, Rodríguez-Molinero et al. proposed a new algorithm that efficiently detects motor fluctuations in patients with PD by using a single inertial sensor located on patient’s waist during gait. Additionally, Pham et al. validated in PD patients and older adults a new step detection algorithm applied to an IMU worn on the lower back, comparing measures with those caught by means of a standardized optoelectronic system. Long-term and objective monitoring of patients’ motor symptoms is a crucial aspect especially for paroxysmal motor disorders, such as l-DOPA-induced dyskinesias and FOG, that are difficult to recognize during standard clinical evaluation of PD patients in the outpatient clinic. For this purpose, Suppa et al. examined an unobtrusive sensory system with an innovative algorithm to automatically detect FOG during gait with high sensitivity and specificity to analyze kinematic gait parameters in relation to the specific motor state (On and Off state of therapy). Accordingly, Palmerini et al. used three wearable inertial sensors to predict FOG through the recognition of specific kinematic gait abnormalities preceding the disorder. Predicting FOG would allow to prevent it by providing external cues that commonly improve gait in patients with PD. In this regard, Ginis et al. monitored cadence of patients with PD through a wearable IMU-based system, investigating the most effective auditory feedback able to support gait and the least heavy one for self-perceived fatigue. Janssen et al. tested the effectiveness of smart glasses delivering 3D augmented reality visual cues to reduce FOG and improve gait parameters measured by IMU, in comparison to conventional 3D transverse bars on the floor and auditory cueing *via* a metronome. Wearable technologies are potentially relevant even in the therapeutic approach in patients with PD by objectively assessing the effect of drugs administration and thus ameliorating the therapeutic strategies for symptomatic improvement in patients with PD. Ruonala et al. used a wearable EMG recording system and two triaxial accelerometers to measure objectively the changes of motor symptoms during l-DOPA challenge test and thus help the selection of patients with PD that could benefit from deep brain stimulation treatment. Finally, overall perspectives of the current technological evolution for objective monitoring of motor symptoms in patients with PD and practical examples of wearable devices application are exhaustively summarized in the perspective article of Matias et al. In conclusion, the achievements of the present research topic allow us to confidently affirm that in the near future, new wireless wearable technologies will likely provide relevant contribution to the early diagnosis and disease progression monitoring of PD, and to design new tailored pharmacological and non-pharmacological therapeutic approaches, thus improving further the clinical management of patients with PD.

## Author Contributions

FI and JC: conception, design, and critical revision of the editorial. AS: conception, design, drafting, and critical revision of the editorial.

## Conflict of Interest Statement

The authors declare that the research was conducted in the absence of any commercial or financial relationships that could be construed as a potential conflict of interest.

## Abbreviations

APAs, anticipatory postural adjustments; DBS, deep brain stimulation; EMG, electromyography; FOG, freezing of gait; IMUs, inertial measurement units; LID, l-DOPA-induced dyskinesia; PD, Parkinson’s Disease.

